# Supplementary material for: Development and Characterization of a Recombinant galT-galU Protein for Broad-Spectrum Immunoprotection Against Porcine Contagious Pleuropneumonia
Source: Int J Mol Sci. 2025 Apr 11;26(8):3634. doi: 10.3390/ijms26083634 (PMC12027175; doi:10.3390/ijms26083634)
Supplement: Supplementary file 1 [file ijms-26-03634-s001.zip › ijms-3511411-supplementary.pdf]

Table S1. Conservation analysis of the galT across serotypes 1–18 of APP.

Nucleotide and amino acid sequence alignment																				
Name	Serotypes Number	1	2	3	4	5	6	7	8	9	10	11	12	13	14	15	16	17	18	19
S4074	1		93.24	94.19	93.24	95.43	95.43	94.1	97.71	96.76	100	98.86	100	94.1	93.9	94.48	97.05	96.86	97.81	97.71
S1536	2	97.42		95.43	99.52	96.38	96.38	95.33	94.95	94.95	93.24	93.71	93.24	95.33	95.14	95.05	94.1	93.9	94.95	94.95
S1421	3	97.99	98.85		95.52	98.29	98.29	99.9	95.9	96.29	94.19	94.57	94.19	99.9	99.71	99.52	96.67	96.48	95.81	95.9
M62	4	97.71	99.71	99.14		96.1	96.1	95.43	95.05	95.05	93.24	94	93.24	95.43	95.24	95.05	94.1	93.9	95.05	95.24
K17	5	97.99	99.43	99.43	99.14		100	98.19	97.05	96.67	95.43	95.52	95.43	98.19	98	97.9	96.76	96.57	96.95	97.05
L20	6	97.99	99.43	99.43	99.14	100		98.19	97.05	96.67	95.43	95.52	95.43	98.19	98	97.9	96.76	96.57	96.95	97.05
Femo	7	97.99	98.85	100	99.14	99.43	99.43		95.81	96.19	94.1	94.48	94.1	100	99.81	99.62	96.57	96.38	95.71	95.81
WF83	8	98.85	97.99	98.57	98.28	98.57	98.57	98.57		98.67	97.71	97.33	97.71	95.81	95.62	95.43	94.76	94.57	99.52	99.62
405	9	98.28	98.57	99.71	98.85	99.14	99.14	99.71	98.85		96.76	97.33	96.76	96.19	96	95.81	95.33	95.14	98.76	98.86
CVJ13261	10	100	97.42	97.99	97.71	97.99	97.99	97.99	98.85	98.28		98.86	100	94.1	93.9	94.48	97.05	96.86	97.81	97.71
D13039	11	98.85	97.99	98.57	98.28	98.57	98.57	98.57	98.28	98.85	98.85		98.86	94.48	94.29	94.1	97.05	96.86	97.43	97.52
56153	12	100	97.42	97.99	97.71	97.99	97.99	97.99	98.85	98.28	100	98.85		94.1	93.9	94.48	97.05	96.86	97.81	97.71
1096	13	97.99	98.85	100	99.14	99.43	99.43	100	98.57	99.71	97.99	98.57	97.99		99.81	99.62	96.57	96.38	95.71	95.81
N273	14	97.42	98.28	99.43	98.57	98.85	98.85	99.43	97.99	99.14	97.42	97.99	97.42	99.43		99.43	96.38	96.19	95.52	95.62
3906	15	98.28	98.57	99.71	98.85	99.14	99.14	99.71	98.28	99.43	98.28	98.28	98.28	99.71	99.14		96.95	96.76	95.33	95.43
HS143	16	98.57	97.71	98.85	97.99	98.28	98.28	98.85	97.42	98.57	98.57	99.14	98.57	98.85	98.28	99.14		99.81	94.86	94.76
A-8514	17	98.28	97.42	98.57	97.71	97.99	97.99	98.57	97.13	98.28	98.28	98.85	98.28	98.57	97.99	98.85	99.71		94.67	94.57
16287-1	18	98.85	97.99	98.57	98.28	98.57	98.57	98.57	99.43	98.85	98.85	98.28	98.85	98.57	97.99	98.28	97.42	97.13		99.71
7311555	19	99.14	98.28	98.85	98.57	98.85	98.85	98.85	99.71	99.14	99.14	98.57	99.14	98.85	98.28	98.57	97.71	97.42	99.71	

The percentage identity of the nucleotide sequences of the *galT* gene is highlighted with a yellow background. In contrast, the percentage identity of the amino acid sequences of the galT protein is displayed against a pale green background.

Table S2. Conservation analysis of the galU across serotypes 1–18 of APP.

Nucleotide and amino acid sequence alignment																				
Name	Serotypes Number	1	2	3	4	5	6	7	8	9	10	11	12	13	14	15	16	17	18	19
S4074	1		98.54	99.21	99.1	98.2	98.2	94.93	98.42	94.93	100	98.54	99.89	98.54	96.4	96.73	99.1	98.65	98.54	98.54
S1536	2	99.32		99.32	98.99	99.66	99.66	96.4	99.89	96.4	98.54	100	98.42	99.77	97.86	98.2	99.21	99.89	100	100
S1421	3	99.32	100		99.66	98.99	98.99	95.72	99.21	95.72	99.21	99.32	99.1	99.1	97.18	97.52	99.89	99.21	99.32	99.32
M62	4	99.66	99.66	99.66		99.1	99.1	95.61	99.1	95.61	99.1	98.99	98.99	98.76	96.85	97.18	99.55	98.87	98.99	98.99
K17	5	98.98	99.66	99.66	99.32		100	96.28	99.77	96.28	98.2	99.66	98.09	99.44	97.52	97.86	98.87	99.55	99.66	99.66
L20	6	98.98	99.66	99.66	99.32	100		96.28	99.77	96.28	98.2	99.66	98.09	99.44	97.52	97.86	98.87	99.55	99.66	99.66
Femo	7	99.32	100	100	99.66	99.66	99.66		96.51	100	94.93	96.4	94.82	96.17	97.64	98.2	95.61	96.28	96.4	96.4
WF83	8	99.32	100	100	99.66	99.66	99.66	100		96.51	98.42	99.89	98.31	99.66	97.75	98.09	99.1	99.77	99.89	99.89
405	9	99.32	100	100	99.66	99.66	99.66	100	100		94.93	96.4	94.82	96.17	97.64	98.2	95.61	96.28	96.4	96.4
CVJ13261	10	100	99.32	99.32	99.66	98.98	98.98	99.32	99.32	99.32		98.54	99.89	98.54	96.4	96.73	99.1	98.65	98.54	98.54
D13039	11	99.32	100	100	99.66	99.66	99.66	100	100	100	99.32		98.42	99.77	97.86	98.2	99.21	99.89	100	100
56153	12	99.66	98.98	98.98	99.32	98.64	98.64	98.98	98.98	98.98	99.66	98.98		98.42	96.28	96.62	98.99	98.54	98.42	98.42
1096	13	98.98	99.66	99.66	99.32	99.32	99.32	99.66	99.66	99.66	98.98	99.66	98.64		97.64	97.97	98.99	99.89	99.77	99.77
N273	14	99.32	100	100	99.66	99.66	99.66	100	100	100	99.32	100	98.98	99.66		96.28	97.07	97.75	97.86	97.86
3906	15	99.32	100	100	99.66	99.66	99.66	100	100	100	99.32	100	98.98	99.66	100		97.41	98.09	98.2	98.2
HS143	16	98.98	99.66	99.66	99.32	99.32	99.32	99.66	99.66	99.66	98.98	99.66	98.64	99.32	99.66	99.66		99.1	99.21	99.21
A-8514	17	99.32	100	100	99.66	99.66	99.66	100	100	100	99.32	100	98.98	99.66	100	100	99.66		99.89	99.89
16287-1	18	99.32	100	100	99.66	99.66	99.66	100	100	100	99.32	100	98.98	99.66	100	100	99.66	100		100
7311555	19	99.32	100	100	99.66	99.66	99.66	100	100	100	99.32	100	98.98	99.66	100	100	99.66	100	100	

The percentage identity of the nucleotide sequences of the *galU* gene is highlighted with a yellow background. In contrast, the percentage identity of the amino acid sequences of the galU protein is displayed against a pale green background.
